# Enteritis

**Published:** 1881-01

**Authors:** G. H. Berns


### ART. VI.—ENTERITIS.

The last and the most dangerous disorder of the intestinal track of the horse is known as Enteritis, or Inflammation of the Bowels.

Enteritis may affect the peritoneal or the mucous coat of the intestines; and in violent cases all the coats may be implicated. When the peritoneal coat is chiefly affected, the disease is known as Sero-enteritis. When the mucous coat is the seat of the trouble, the term Muco-enteritis is employed; but as it is almost impossible, in acute cases of inflammation of the bowels, to distinguish between the two forms, from the symptoms present, the term Enteritis is generally used—meaning either one or the other.

Any portion of the intestinal tube may be involved; but, according to Williams, the cœcum and colon are the parts most frequently affected. In two cases which terminated fatally under my care, the post-mortem examinations revealed that the small intestines were the seat of the trouble.

Acute enteritis proves rapidly fatal in very many cases, and often destroys life in a few hours.

Sub-acute muco-enteritis, on the other hand, is a very different form of trouble. It produces diarrhœa or dysentery, and, if allowed to go on unchecked, will cause death from exhaustion after weeks or even months of suffering. When we speak of enteritis, the acute form is generally meant, and that is the form we will consider here.

*Causes.*—Exposure to damp and cold, or washing a horse's body with cold water when warm, causing contraction of the cutaneous blood-vessels, thereby disturbing the circulation, limiting the amount of blood to the surface of the body, and producing engorgement of the deeper seated vessels, and congestion and inflammation of the parts that they supply. Now, if any particular organ or sets of organs are weakened from any cause, such organs are most likely to suffer, and if, in connection with exposure to cold and damp, a horse's bowels have been rendered predisposed by previous attacks of spasmodic colic, tympanites, mesenteric abscesses, intestinal concretions, diarrhœa or constipation, such exposure would be the indirect cause of inflammation of the bowels.

Spasmodic or wind colic, if not checked, may, and often does, terminate in enteritis. Irritating remedies, such as turpentine, ammonia, pepper, etc., improperly prepared, or given in overdoses for the relief of spasmodic or flatulent colic, frequently prove to be the direct causes of enteritis.

*Symptoms.*—As the symptoms of this disease closely resemble those of spasmodic colic, and as enteritis is frequently accompanied by tympanic symptoms, it is a matter of considerable difficulty to make a correct distinction between these three important disorders. Enteritis, unless preceded by spasmodic or flatulent colic, is usually ushered in by a chill. The pain is constant and of the severest kind, causing the animal to throw himself about in his stall in the most violent manner, or he may stand for hours in the same position, pawing the floor with one or both feet alternately, or he may stand perfectly quiet, as if afraid to move, now and then turning his head dolefully to his flank.

The pulse is increased in frequency, and thready, and soon runs up to from 80 to 120, and is then hardly perceptible. The temperature ranges from 104 to 106, or even 107. Respiration is short and quick, nostrils widely dilating at every inspiratory movement. The expression of the face is extremely anxious; cold sweats bedew the body. The mouth is dry, and the tongue seems parched and contracted. The visible mucous membranes present a deep red color, and pressure in the abdominal region causes the animal to groan with pain. The eyes acquire a wild, unnatural stare; the pupils dilate; the mouth now becomes cold, the breath cold and even fœtid; and in this condition the patient generally drops down, and dies after a few feeble struggles. In some cases the symptoms of agony and pain subside suddenly; the horse may stand quietly in his stall, and if food is offered him, may make a feeble attempt at feeding, and, to the inexperienced, the patient would seem to be improving, when in reality mortification has seized the inflamed bowel and pain is no longer felt. In this condition he may die within a few minutes, or may linger for several hours or even days; but death is sure to follow. The history of the case, particularly the manner of attack, the state of the pulse, the pain on pressure in the abdominal region, the peculiar, anxious expression of the countenance, the dryness of the mouth, and the elevation of temperature, may be considered as diagnostic symptoms of enteritis, and distinguish it from all other forms of abdominal trouble.

*Treatment.*—As the disease usually terminates fatally in a short period of time, the treatment must be very prompt. Opium, given in full doses per mouth, or atropia and morphia combined and administered sub-cutaneously, together with the judicious use of enemas, and strong counter-irritants to the abdomen, are about all that can be done. If the case is seen early, moderate blood-letting proves beneficial.

G. H. BERNS, D. V. S.

